# Açai (*Euterpe oleracea* Mart.) Seed Extract Induces Cell Cycle Arrest and Apoptosis in Human Lung Carcinoma Cells

**DOI:** 10.3390/foods7110178

**Published:** 2018-10-26

**Authors:** Raquel Martins Martinez, Deborah de Almeida Bauer Guimarães, Camila Ramos Berniz, Joel Pimentel de Abreu, Ana Paula Machado da Rocha, Roberto Soares de Moura, Angela Castro Resende, Anderson Junger Teodoro

**Affiliations:** 1 Nutritional Biochemistry Core, Laboratory of Functional Foods, Universidade Federal do Estado do Rio de Janeiro (UNIRIO), Avenida Pasteur 296-Urca, Rio de Janeiro 22290-240, Brazil; raquelm.martinez@hotmail.com (R.M.M.); deborahbauervp@gmail.com (D.d.A.B.G.); camila.rberniz@hotmail.com (C.R.B.); pimenabreu@gmail.com (J.P.d.A.); 2 Department of Physiological Sciences, Institute of Biomedicine Universidade Federal do Estado do Rio de Janeiro (UNIRIO), Rua Frei Caneca, 94, Rio de Janeiro CEP 20211-040, Brazil; ana.rocha@unirio.br; 3 Department of Pharmacology, Institute of Biology, Universidade Estadual do Rio de Janeiro (UERJ), Av 28 de Setembro, n° 87, Rio de Janeiro CEP 20551-030, Brazil; robertosoaresdemoura@gmail.com (R.S.d.M.); angelacr@uerj.br (A.C.R.)

**Keywords:** açai, antioxidant, bioactive compounds, lung cancer

## Abstract

Açai fruit has been studied for its antioxidant properties, with positive feedback against many diseases, including cancer. Although açai seeds are not edible, their composition has been studied in order to find new applications and reduce garbage generation. This study aimed to evaluate the cytotoxic effects and impacts on the cell cycle and apoptosis of açai seed extract (ASE) on human lung carcinoma cell line (A549). Antioxidant activity of açai seed extract (ASE) was measured by DPPH assay, Trolox Equivalent Antioxidant Capacity (ABTS/TEAC), Ferric Reducing Ability (FRAP) and Oxygen radical absorbance capacity (ORAC) assays. Human lung carcinoma cell viability (A549) was monitored by MTT assay method and the effects on cell cycle and apoptosis were measured by flow cytometry. The results indicate high antioxidant activity in ASE and high values of total phenolic compounds (37.08 ± 8.56 g gallic acid/100 g). The MTT assay showed a maximum decrease (72.07%) in the viability of A549 cells after 48 h treatment with ASE (200 µg/mL). Flow cytometer analysis revealed that ASE increased the percentage of cells in G0/G1 phase and promoted a high increase of apoptotic cells when compared to the untreated cells. The present study suggests that ASE has a high antioxidant capacity and may have a protective effect against lung cancer.

## 1. Introduction

Cancer is a worldwide health concern because of its incidence and mortality. The most common type is lung cancer, with an annual incidence rate estimated at 1.82 million [1]. The existing therapeutic strategies for lung cancer include surgery, radiotherapy, chemotherapy and physical therapy. The survival rate of non-small-cell lung cancer patients is still less than 1% [2]. Smoking and exposure to pollutants are considered the main causes of lung cancer, and, in contrast, nutrition has been thought to be a preventable cause. High consumption of fruits and vegetables can be an important element of primary prevention [3].

Berries are rich sources of bioactive nutrients and essentially experimental data suggest that the regular consumption of berries or berry-derived natural products can help prevent and treat a wide range of pathologies [4]. As a strong antioxidant berry, açai fruit has been studied by its properties, with positive feedback against chronic non-communicable diseases and different types of cancer [5,6,7].

*Euterpe oleracea* Mart. is a tropical palm, typically found in the Amazon region. Its fruit is widely known as açai, whose consumption is identified as a common habit in the North and Northeast of Brazil. The international food market has shown higher interest in this fruit over recent decades, increasing Brazilian production and exportation. In addition to the antioxidant properties, açai also has an interesting nutritional composition of fatty acids, fibers and micronutrients. For this reason, it is considered to be a functional food, currently consumed not only as juice but also as energy drinks, ice cream and even as a supplement powder [8].

Each açai fruit contains one light brown seed that accounts for about 90% of the fruit’s diameter (1–2 cm) and up to 90% of its weight (0.7–1.9 g). Due to the production increase, there is also a higher residual amount of seeds. Only a small portion of the seeds are utilized as pig food or, when rotten, for making a very rich potting soil for plantations or home gardens [9]. Although açai seeds are not edible, their composition has been studied in order to find new applications and reduce garbage generation [10,11].

In this context, this study aimed to evaluate the cytotoxic effects and impacts on the cell cycle and apoptosis of açai seeds extract (ASE) on human lung carcinoma cell line (A549).

## 2. Materials and Methods

### 2.1. Hydro-Alcoholic Açai Seed Extract (ASE)

*Euterpe oleracea* Mart. fruits were obtained from the Amazon Bay (Pará, Brazil), washed in tap water, and the skins were separated from the stones. Approximately 200 g of stones were boiled in 400 mL of water for 5 min, ground for 2 min and then boiled again for another 5 min. The decoction was allowed to cool at room temperature, extracted with 400 mL of ethanol, shaken for 2 h and then kept in the dark bottles inside a refrigerator (4 °C) for 10 days. After the maceration period, the hydro-alcoholic extracts of açai were filtered through Whatman n°1 filter paper and the ethanol was evaporated under low pressure at 55 °C. The extract was then lyophilized and frozen at −20 °C until use. Usually, 100 g of stones yields 5 g of lyophilized extract [12].

### 2.2. Antioxidant Activity Assays and Total Phenolic Compounds

A standard solution of açai extract (ASE) (2.5 mg/mL) and a curve with different concentrations (0–300 L of standard solution) were used for antioxidant assays. ASE was resuspended in water for all analysis.

The extracts were measured for antioxidant activity by Ferric Reducing Ability (FRAP) according to Bauer et al. [13]. Aliquots of 2.7 mL of TPTZ reagent (ferric 2,4,6-tripyridyl-s-triazine) (10 mM) reagent (ferric 2,4,6-tripyridyl-s-triazine) were mixed with different concentrations of ASE. After 30 min at 37 °C, the absorbance was read at 595 nm. The antioxidant capacity (FRAP) was expressed as Fe^3+^ equivalents (mol ferrous sulfate/g of ASE). For DPPH assay, aliquots of ASE were mixed with 2.5 mL DPPH methanolic solution (0.06 mM) and allowed to react for half an hour, in the dark. Measurements were performed at 515 nm applying a Turner^®^ 340 spectrophotometer (Barnstead/Thermolyne, Dubuque, IA, USA). The analysis was performed in triplicate and the decline in the DPPH radical absorbance concentration caused by the samples was measured. The results are expressed as % of reduction of radical.

The TEAC+ cation was prepared by mixing a TEAC stock solution (7 mM in water) with 2.45 mM potassium persulphate. This mixture was allowed to stand for 16 h at room temperature until the reaction was completed and the absorbance was stable. The antioxidant capacity assay was carried out by the improved Trolox Equivalent Antioxidant Capacity (ABTS/TEAC) method of Bauer et al. [13]. TEAC solution (2.5 mL) was added to samples or commercial antioxidant (Trolox) and mixed thoroughly. Absorbance was recorded at 734 nm for 6 min. Results were calculated as mol Trolox/μg of ASE. The Oxygen radical absorbance capacity (ORAC) procedure used an automated plate reader (SpectraMax i3x, Molecular Devices, San Jose, CA, USA) with 96 well plates [13,14]. Experiments were conducted in phosphate buffer pH 7.4 at 37 °C. Peroxyl radical was generated using 2,2′-azobis (2-amidino-propane) dihydrochloride, which was prepared fresh for each run. Fluorescein was used as the substrate. Fluorescence conditions were as follows: excitation at 485 nm and emission at 520 nm. The standard curve was linear between 0 and 50 mM Trolox. Results are expressed as mEq Trolox.

### 2.3. Cell Culture and Treatment Protocol

Human lung carcinoma cell line (A549) was obtained from the Rio de Janeiro Cell Bank which certified their identity and quality (INMETRO, Rio de Janeiro, RJ, Brazil). A549 cell line was plated in 25 cm^2^ tissue culture flasks (5.0 × 10^6^ cells/flask) and maintained routinely in Dulbecco’s Modified Eagle’s Medium-high glucose (DMEM) supplemented with 10% fetal bovine serum (FBS) and 1% penicillin (PS), pH 7.4, under 5% CO_2_ atmosphere. Stock flasks were grown to 70% confluence and subcultured routinely. The medium renewal was done 3 times weekly.

### 2.4. Cell Viability

The status of cancer cell line viability was determined by MTT (3-(4,5-dimethylthiazol-2-yl)-2,5-diphenyltetrazolium bromide; thiazolyl blue) assay (Sigma, New York, NY, USA) as first described by Mosmann [13]. The cells were incubated with ASE resuspended in DMEM (1.25–200 µg/mL) for 48 h (6 wells for each sample). Six wells were included for control (DMEM). The cell proliferation inhibition rate (CPIR) was calculated using the following formula: CPIR = (1 − average value of experimental group/average value of control group) × 100%.

### 2.5. Cell Cycle Analysis

Cells were rinsed briefly with calcium and magnesium-free phosphate-buffered saline and detached with trypsin at room temperature. After centrifugation, the cells were washed twice with PBS; cells were resuspended in 500 µL of ice-cold Vindelov solution containing 0.1% Triton X-100, 0.1% citrate buffer, 0.1 mg/mL RNAse, and 50 mg/mL propidium iodide (Sigma Chemical Co., St. Louis, MO, USA). After 15 min of incubation, cell suspension was analyzed for DNA content by using a FACSCalibur flow cytometer (Becton Dickinson, Mountain View, CA, USA). The relative proportions of cells with DNA content indicative of apoptosis (<2 n), G0/G1 diploid (2 n), S (phase >2 n but <4 n), and G2/M phase (4 n) were obtained and analyzed using CellQuest and WinMDI 2.9. The percentage of the cell population at a particular phase was estimated with FlowJo software v.7.6.5 (Ashland, OR, USA). Cell dissociation procedure does not affect fluorescence under the experimental conditions that were used in this study or in any other studies of which we are aware. Nuclei of viable cells were gated according to FL-2W × FL2-A relation. Two concentrations were tested (50 and 100 µg ASE/mL) besides control (DMEM).

### 2.6. Apoptosis Assay

Cells were resuspended in 400 μL of binding buffer containing 5 μL of annexin V FITC and 5 μL propidium iodide (Apoptosis Detection Kit II, BDBiosciences, Mountain View, CA, USA ) for 15 mins at room temperature. Annexin V binding was evaluated by flow cytometry FACSCalibur, Becton Dickinson, Mountain View, CA, USA), and after acquisition of 20,000 events the data were analyzed in Cell Quest software. Two concentrations were tested (50 and 100 µg ASE/mL) besides control (DMEM).

### 2.7. Statistical Analysis

Results are presented as mean with the corresponding standard deviation of experiments done in triplicates. Data were analyzed with the statistical software GraphPad Prism (version 5.04, GraphPad Software, San Diego, CA, USA). One-way analysis of variance (ANOVA) test with the posttest of Tukey at a confidence level of 95% was used for all assays.

## 3. Results and Discussion

### 3.1. Antioxidant Activity and Total Phenolic Compounds

The analysis of the aqueous fraction residue from ASE (2.5 mg/mL) by high- performance liquid chromatography (HPLC) and matrix-assisted laser desorption/ionization (MALDI-TOF) mass spectrum was reported by our group [9,14,15,16,17]. The HPLC analysis of ASE revealed that it is composed by proanthocyanidins (88% of the total area) and in minor extent catechin and epicatechin. The chemical and spectrometric analysis revealed that ASE is composed predominantly of polymeric procyanidins, heteropolymers with one gallocatechin unit and, a minor extent, of galloylated procyanidins [14].

Since the antioxidant capacity of food is determined by a mixture of different bioactive compounds with different action mechanisms, among which synergistic interactions, it is necessary to combine more than one assay in order to determine, in vitro, the antioxidant capacity of fruits and vegetables (Table 1). ASE (100 µg/mL) showed a potent reduction in DPPH radical (92.05 ± 2.56%) and ABTS method (566.01 ± 43.24 μM TEAC/μg). Through FRAP and ORAC methods, the respectively results obtained were 8.98 ± 0.35 mmol Fe2 + Eq/g of extract and 16679.17 ± 4879.81 µM TE. These results pointed that the extract produced has a high antioxidant potential to use in food and health products.

In a review of the properties of açai, Yamaguchi et al. [8] associate a high antioxidant activity against the DPPH radical, anion superoxide, peroxyl radicals, hydroxyl radicals and inhibition of oxidation of liposomes to this fruit. The same study presents a high number of bioactive substances described in açai, of which approximately 31% consists of flavonoids, followed by phenolic compounds (23%), lignoids (11%) and anthocyanins (9%). A total of 37.08 ± 8.56 g/100 g gallic acid was quantified in ASE, indicating a high antioxidant activity of ASE and considered compatible with previous studies of the same fruit [9,10]. Antioxidants are thought to help protect the body against the damaging effects of free radicals and the chronic diseases associated with the aging process. Also, the benefits of bioactive compounds for human health are also known as anticancer, antimutagenic, antimicrobial, anti-inflammatory, and neuroprotective [18].

Foods rich in polyphenols, considered to have high antioxidant power, mainly those in the class of anthocyanins, are increasingly being used in the prevention of diseases. Acai (Euterpe oleracea) is a fruit that stands out for presenting this property, and even though Brazil is the largest producer of this fruit, the foreign market has been investing in its exportation for use in both the food and pharmaceutical industries [19,20].

### 3.2. ASE Effects on Lung Cancer Cells Line (A549)

**Figure 1:**
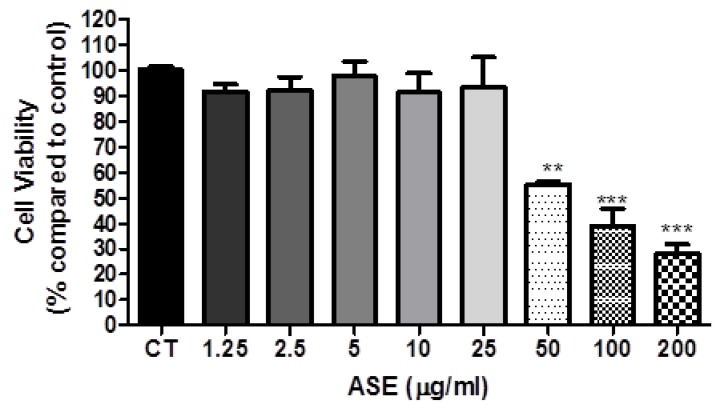
Cell viability of A549 cells treated with açai seed extract (ASE). The cells were treated with ASE (1.25–200 µg/mL for 48 h, and the MTT assay was done. The results are expressed as % compared to the control, and expressed as mean 6 standard deviations of 3 independent experiments, each performed with at least 3 replicates. (**, ***) indicates significant differences from the control group (** *p* < 0.01, *** *p* < 0.001).

After 48 h of treatment with ASE, the cell viability of lung cancer cells line A549 on MTT assay decreased considerably when compared to control (Figure 1). The effect was dose-dependent, with a maximum reduction of 72.07% by 200 µg/mL of extract. Similar results were related by Barros et al. [10], where açai seeds extract showed inhibitory effect on the growth of different human tumor cell lines (MCF-7, NCI-H460, HeLa and HepG2), being more effective for the cervical carcinoma cell line. It was possible to identify the antiproliferative pathways by which açai acts by reducing proliferating cell nuclear antigen (PCNA), Ki-67 and p63 [6,7,21]. These proteins are involved in tumor development, survival and metastasis of different tumors [22,23,24].

The absence of toxicity of açai was reported in previous studies after testing açai in experimental models, and no significant differences were reported [25]. DNA damage induced by antitumor medication was evaluated in 3 studies, and no genotoxic effects were observed after açai administration by gavage [26,27,28]. In a study done by Schauss et al., açai did not cause mutagenic effects [28]. In the same way, Marques et al. evaluated the genotoxic potential of açai in rat cells and showed that on both cytogenetic tests, no significant genotoxic effects were observed at the three tested dosages of açai [27].

**Figure 2:**
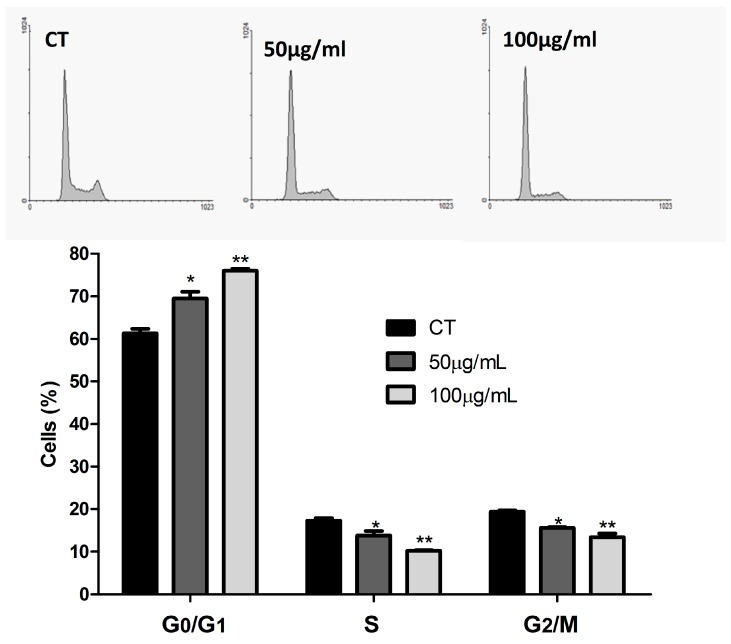
Effect of açai seed extract (ASE) on cell cycle progression in A549 cells after 48 h of treatment. The phases of the cell cycle are illustrated at control (CT) and treated with 50 and 100 µg/mL of ASE. The experiment is expressed as mean ± standard deviation. Significant differences between untreated cells (CT) and treated with açai seed extract (ESA) (50–100 µg/mL) were compared by one-way analysis of variance (ANOVA) followed by Tukey multiple comparison post-hoc test (* *p* < 0.05. ** *p* < 0.01).

The cell cycle on cancer cells is affected by a disorder of regulation mechanisms, commonly associated with proteins (cyclins) and inhibitor genes (p16, p27, p21, p53), which results in uncontrolled cell proliferation [29]. The cyclin-dependent kinases (CDKs) are rational targets for cancer therapy. Their expression is often perturbed in malignancy, and their inhibition can induce apoptosis. Checkpoint integrity is often lost as a result of inactivation of CDKIs or overexpression of cyclins. For example, loss of p16 function is associated with melanoma, lung, breast, and colorectal tumors. Thus, targeting CDKs could restore cell-cycle checkpoints and may slow growth or induce apoptosis [30]. Many bioactive compounds have been associated with cell cycle arrest of cancer cells through downregulation of cyclins [4] and other beneficial effects, such as phase I carcinogen-metabolizing enzymes inhibition, phase II detoxification enzymes induction, enhance of immune system, and modulation of circulating hormone concentrations [3]. Figure 2 shows the ASE effect on the cell cycle of lung cancer cells line (A549) after a 48 h treatment. Cells at G_0_/G_1_ phase were increased, reducing the following phases S and G_2_/M. Those results show a possible regulation of the cell cycle by the influence of bioactive compounds present in the extract.

**Figure 3:**
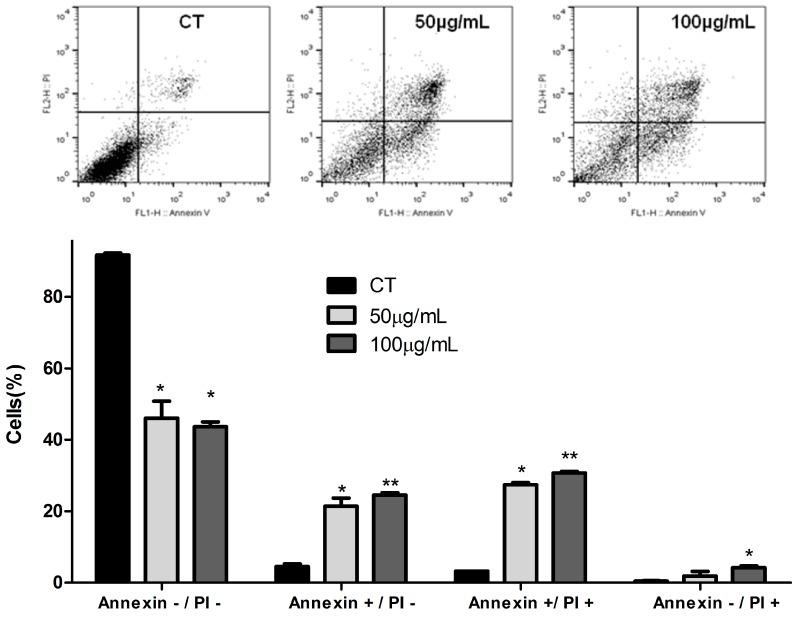
Effect of açai seed extract (ASE) on stages of death process in human lung carcinoma cells (A549) after 48 h. Results are expressed as a percentage of total cells. The experiment is expressed as mean ± standard deviation, with significant differences between untreated cells (CT) and treated with açai seed extract (ASE) (50 and 100 µg/mL) were compared by One-way ANOVA with the post-test of Tukey (* *p* < 0.05, ** *p* < 0.01).

The literature also suggests that defects along apoptotic pathways play a crucial role in carcinogenesis, and that many new treatment strategies targeting apoptosis are feasible and may be used in the treatment of various types of cancer [31]. The incubation with the extract showed a significant effect on apoptosis assay. Figure 3 shows that cells treated with 50 µg/mL and 100 µg/mL ASE for 48 h resulted in a significant increase in the percentage of apoptotic cells (early and later apoptosis) and a significant decrease in viable cells compared with untreated cells (control). Pozo-Insfran et al. [32] demonstrated that polyphenolics present in açai reduced the proliferation of HL-60 leukemia cells through caspase-3 activation in a dose-and-time-dependent manner. Choi et al. [7] have also shown a protective effect of açai against colon carcinogenesis induced by azoxymethane/dextran sulfate sodium in rats. This result was associated with a reduction of COX-2, TNF-α, IL-1β and IL-6 expression levels in macrophages and the mouse colon, suppression of Bcl-2 and PCNA, and activation of the mitochondrial proapoptotic pathway.

Another possible mechanism of action of açai was previously demonstrated. Dias et al.’s [33] results demonstrate that açai polyphenolic extract induced cytochrome c, cleaved caspase-3, and decreased the antiapoptotic PARP-1. The mechanisms involved in colon cancer model cell growth suppression include protection against reactive oxygen species (ROS) production, down-regulation of NF-kB (factor nuclear kappa B) and NF-kB-target VCAM-1 (vascular cell adhesion molecule 1) and ICAM-1 (intercellular adhesion molecule 1), and the downregulation of Sp prooncogenic transcription factors and Sp-targets VEGF (vascular endothelial growth factor), Bcl-2, and survivin. Future research is needed to better understand the efficacy of ASE extract because its antimutagenic and antioxidant activities may improve human health.

## 4. Conclusions

The açai fruit has been highlighted for its significative types of bioactive compounds and its benefits. Despite the majority of studies using the fruit pulp, the seed showed a great antioxidant potential that should be considered. Taken together, the results indicated that ASE consumption could reduce the proliferation of human lung cancer cells, an effect potentially mediated by the antioxidant activity and modulation cell cycle and apoptosis rate. However, before the applicability of ASE as a functional food can be determined, further research is needed to elucidate the mechanism by which ASE anthocyanins act to selectively modulate cancer and to determine the optimal level of consumption that promotes satisfactory effects.

## Figures and Tables

**Table 1:** Antioxidant activity and total phenolic compounds of Açai Seed Extract (ASE).

Parameters	Açai Seed Extract (ASE)
Total phenolic compound (acid gallic equivalent g/100 g of ASE)	37.08 ± 8.56
DPPH (% of reduction)	92.05 ± 2.56
ABTS (μM trolox equivalent antioxidant capacity/μg of ASE)	566.01 ± 43.24
FRAP (mmol Fe2 + Eq/g of ASE)	8.98 ± 0.35
ORAC (μM equivalent of Trolox)	16679.17 ± 4879.81
